# A Case of Emphysematous Osteomyelitis of the Midfoot: Imaging Findings and Review of the Literature

**DOI:** 10.1155/2014/616184

**Published:** 2014-06-09

**Authors:** Marcela Mautone, Jessica Gray, Parm Naidoo

**Affiliations:** ^1^Monash Medical Centre, Monash Health, 246 Clayton Road, Clayton, Melbourne, VIC 3168, Australia; ^2^University of Auckland, Private Bag 92019, Auckland 1142, New Zealand; ^3^Department of Radiology, Monash Health, 246 Clayton Road, Clayton, Melbourne, VIC 3168, Australia; ^4^Faculty of Medicine, Monash University, Melbourne, VIC 3800, Australia

## Abstract

Emphysematous osteomyelitis is a rare but potentially fatal condition that must be considered whenever intraosseous gas is identified on imaging. The organisms implicated in most cases of emphysematous osteomyelitis are anaerobes or members of the *Enterobacteriaceae* family. Significant comorbidities, such as malignancy and diabetes mellitus, frequently predispose to this condition, and high mortality rates have been reported. The radiologist must be aware of the implications of identifying intraosseous gas in order to facilitate early diagnosis and expedite management. We report a unique case of a 58-year-old male with diabetes mellitus who presented with emphysematous osteomyelitis of the midfoot and necrotising fasciitis of the ipsilateral distal lower limb. Specimen cultures in this case revealed a pure growth of Group G *Streptococcus*.

## 1. Introduction

The presence of intraosseous gas in the absence of direct communication between bone and air, such as a compound fracture or recent surgery, is highly suggestive of emphysematous osteomyelitis [1, 2]. In 1981, Ram et al. were first to describe the radiological finding of intraosseous gas as a sign of osteomyelitis on computed tomography (CT) [3]. Subsequently, in 1983, Patton and colleagues described intramedullary gas within the femur on plain film [4]. We report a case of a 58-year-old male with diabetes mellitus who presented with emphysematous osteomyelitis of the midfoot and necrotising fasciitis of the ipsilateral distal lower limb and a pure growth of Group G* Streptococcus* from specimen cultures.

## 2. Case Presentation

A 58-year-old male presented to our institution with fever and a painful and erythematous right lower limb. His medical history consisted of poorly controlled diabetes mellitus, chronic alcohol abuse, heavy smoking, hypertension, splenectomy for previous trauma, and right transmetatarsal amputation for a nonhealing ulcer in the same foot in the previous year. The wound from his previous forefoot amputation had only partially healed, despite the amputation having been performed several months earlier, and ten days prior to his presentation the patient sought medical attention for increasing purulent discharge from the stump wound. He was commenced on oral amoxicillin/clavulanic acid by his general practitioner.

On admission to hospital, the patient was tachycardic and febrile (38°C). Lower extremity examination revealed erythema, oedema and tenderness of the right midfoot extending to the ankle, and a gangrenous area on the dorsal aspect of the foot with associated putrid purulent discharge. Documented examination findings at presentation did not report crepitation. Posterior tibial and dorsalis pedis pulses were impalpable. Laboratory findings included leukocytosis (28.9 × 10^9^/L with 83% neutrophils), hyperglycemia (16.8 mmol/L), elevated C-reactive protein (244 mg/dL, to convert to nanomoles per litre, multiply by 9.524), and haemoglobin A1c (9.1%). Renal and liver function tests were within normal range. Two sets of blood cultures and a sample of the purulent exudate for microbiology were obtained prior to commencement of empirical intravenous piperacillin and tazobactam.

**Figure 1:**
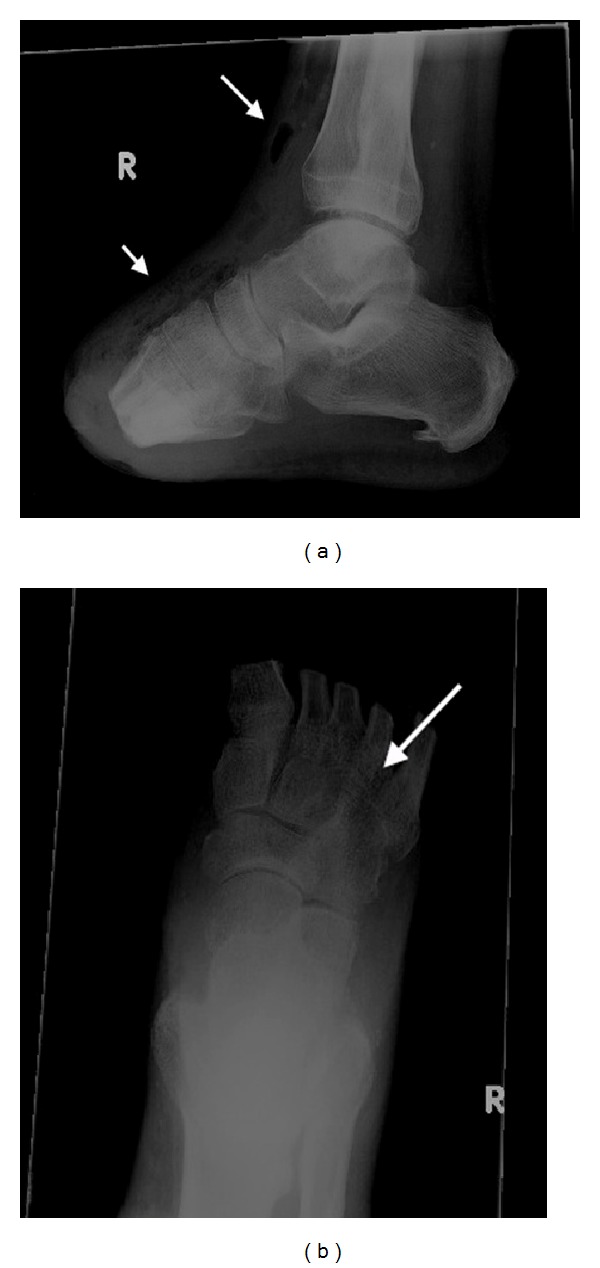
Right foot radiographs reveal extensive soft tissue gas in the dorsum of the foot ((a), short arrow) and anterior to the distal tibia ((a), long arrow). Anteroposterior view demonstrates discrete radiolucencies in the metatarsals remnants ((b), arrow) and midtarsal bones. Previous transmetatarsal amputation is noted.

**Figure 2:**
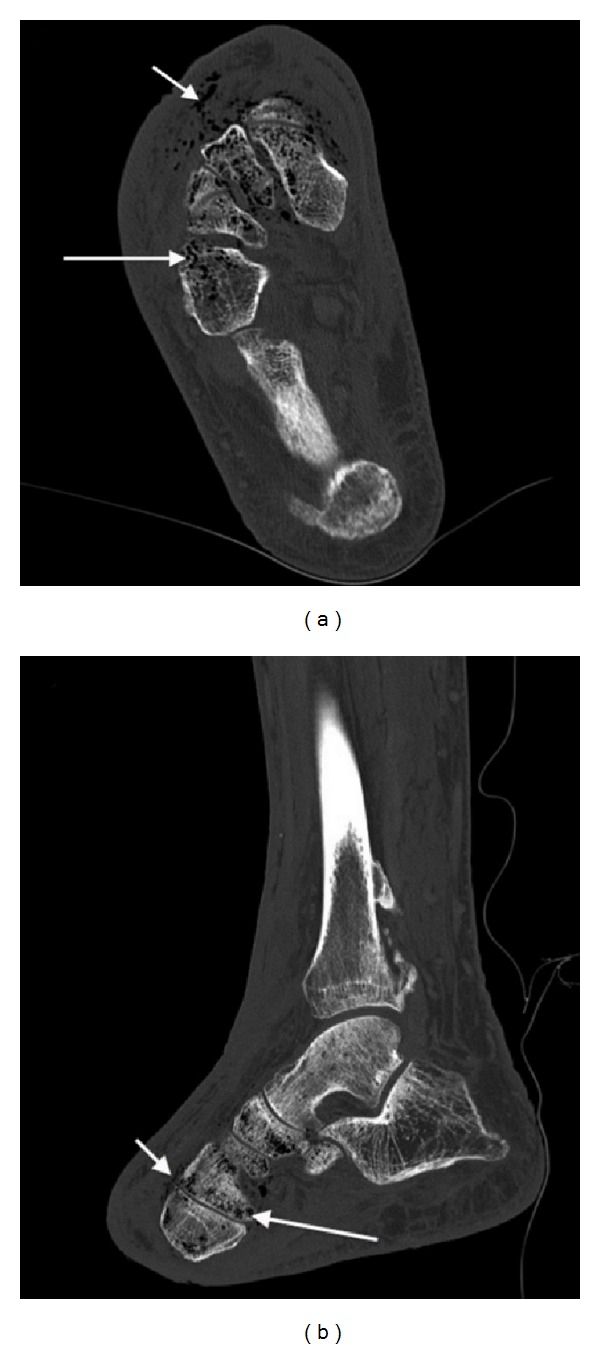
Computed tomography demonstrates gas within the midtarsal bones (long arrow, (a) and (b)) and head of talus and gas in the soft tissues surrounding the midfoot (short arrow, (a) and (b)).

**Figure 3:**
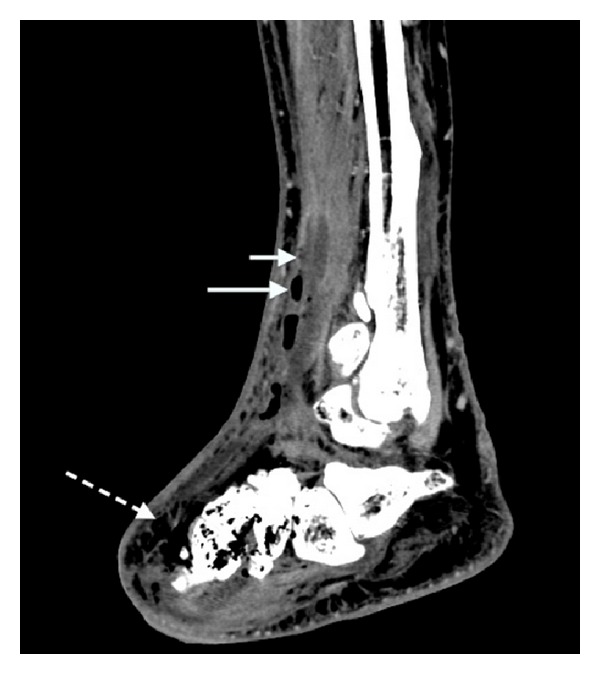
Computed tomography demonstrates soft tissue abscess formations anterior to the distal tibia with fluid (short arrow) and air-fluid levels (long arrow). Gas locules in the midfoot bones and extensive subcutaneous gas involving predominantly the dorsum of the foot (dashed arrow) and anterior aspect of the ankle are evident.

Right foot X-ray revealed soft tissue gas in the dorsum of the foot and anterior aspect of ankle, suggestive of necrotising fasciitis, with the suspicion of gas in the metatarsals and midtarsal bones (Figure 1). Subsequent computed tomography confirmed the presence of multiple locules of gas within the metatarsals remnants, midtarsal bones, and head of the talus (Figure 2), indicative of emphysematous osteomyelitis. CT also revealed loculated fluid collections in the soft tissues anterior to the distal tibia with air-fluid levels (Figure 3), concerning for abscesses. Extensive subcutaneous emphysema involving the dorsum of the foot and anterolateral aspect of the ankle was again noted on CT (Figures 2 and 3).

The patient underwent urgent right below-knee amputation. Gram stain of the stump wound and intraoperative bone specimen revealed abundant gram-positive cocci and few gram-negative bacilli, and culture revealed a pure growth of Group G* Streptococcus*. Blood cultures remained negative. Antibiotic therapy was adjusted to intravenous meropenem and lincomycin and it was continued for a total of six days, followed by oral clindamycin for a further week. No complications were seen on follow-up at six weeks.

## 3. Discussion

Emphysematous osteomyelitis is a rare but serious condition. Since Ram and colleagues' first description of emphysematous osteomyelitis in 1981 [3], the prevalence has been very low, and only 25 cases had been reported in English literature to 2011 [5]. All reported cases have been in either one or more of the following locations: thoracic and lumbar vertebrae, sacral bones, femur, pelvis, tibia, or fibula [5]. To the best of our knowledge, emphysematous osteomyelitis of the tarsals and metatarsals has not previously been described. Underlying comorbidities known to compromise immune function, such as diabetes mellitus and malignancy, are commonly associated with the disease [1]. In our patient, immunosuppression due to poorly controlled diabetes mellitus, splenectomy, and alcohol abuse likely played an important role in the development of this serious infection.

The mechanism of infection is most often by hematogenous dissemination [5]. Cases of emphysematous osteomyelitis from the spread of an intra-abdominal infection, intra-abdominal or spinal surgery, or from a soft tissue or skin infection have also been reported [5, 6]. In this case, a partially open wound from previous transmetatarsal amputation was the probable portal of infection, progressing to necrotising fasciitis and subsequently emphysematous osteomyelitis.

Causative organisms include a range of both aerobic and anaerobic bacteria. Most commonly cultured organisms from both mono- and polymicrobial reported cases of emphysematous osteomyelitis include an anaerobe or a member of the Enterobacteriaceae family [5]. In our case, Gram stain suggested a polymicrobial infection with abundant gram-positive cocci, and the culture indicated that Group G* Streptococcus* was the predominant organism. Growth of the gram-negative bacilli seen on Gram stain, and potentially anaerobes, may have been suppressed by prior antibiotic therapy. Interestingly, this is the first reported case of emphysematous osteomyelitis to isolate Group G* Streptococcus*. Lancefield Group G streptococci have been identified as part of the normal microbial flora of the pharynx, skin, vagina, and gastrointestinal tract [7].It is known to cause a spectrum of invasive infections, including necrotising fasciitis and osteomyelitis, usually in patients with significant underlying disease such as diabetes mellitus and malignancy [8]. Therefore, given the virulence of this organism, it would seem reasonable to conclude that our patient developed necrotising fasciitis and emphysematous osteomyelitis predominantly, if not entirely, due to Group G* Streptococcus* infection.

The radiological differential diagnosis for the presence of intraosseous gas includes osteonecrosis, bone malignancy, postbiopsy, penetrating wounds, compound fractures, and lymphangiomatosis of the bone [1, 2, 9]. Gas may also accumulate anywhere in the body where substantial negative pressure develops [10]. Intravertebral gas may be seen when negative pressure develops in the intervertebral disc as a manifestation of disc degeneration [9]. On the other hand, extensive intravertebral gas associated with bone oedema and/or fluid collections should raise suspicion of emphysematous osteomyelitis [5].

Radiological examination played a critical role in determining the nature and extent of infection in our patient and enabled the implementation of an aggressive treatment plan. Plain films suggested the rare finding of intraosseous locules of air as well as significant soft tissue gas, prompting the diagnosis of a serious infection, initially considered to represent necrotising fasciitis. CT not only confirmed the X-ray findings, but also revealed the extent of intraosseous gas and depicted soft tissue abscesses. Surgical intervention is the cornerstone of treatment of both emphysematous osteomyelitis and necrotising fasciitis [1, 8]. Treatment in our patient required below-knee amputation in an attempt to remove all infected bone and soft tissue. Ultimately, amputation, surgical debridement, and intravenous antibiotic therapy proved successful in controlling the spread of the infection and in preventing further tissue loss.

Emphysematous osteomyelitis is associated with high mortality (32%) and significant morbidity, especially in diabetic patients [5]. Early diagnosis and immediate treatment are crucial in preventing the potentially devastating consequences of the disease. Therefore, the radiologist must be aware that intraosseous gas is a rare but alarming sign for the potentially fatal condition of emphysematous osteomyelitis.
